# Evaluation of the modulating effect of epidermal growth factor receptor inhibitor cetuximab in carbon-tetrachloride induce hepatic fibrosis in rats

**DOI:** 10.1016/j.bbrep.2024.101689

**Published:** 2024-03-21

**Authors:** Mirza Alimullah, Asif Ul Haque Shuvo, Ishrat Jahan, Iffat Nowshin Ismail, S.M. Mufidul Islam, Mahnaj Sultana, Mahmudur Rahman Saad, Sabbir Raihan, Ferdous Khan, Md. Ashraful Alam, Nusrat Subhan

**Affiliations:** Department of Pharmaceutical Sciences, North South University, Bangladesh

**Keywords:** Cetuximab, Fibrosis, Superoxide dismutase, Inflammation, Transforming growth factor-*β*

## Abstract

Liver fibrosis, developed in almost all chronic liver injuries. Epidermal growth factor receptors (EGFR) have been thought to contribute to cirrhosis and liver fibrosis. Therefore, using a rat model of carbon tetrachloride (CCl_4_)-induced liver fibrogenesis, we investigated the preventive effects of cetuximab, an inhibitor of the EGF receptor (EGFR). Ameliorative effects of cetuximab were examined in rats, brought on by biweekly doses of 50 mg/kg of carbon tetrachloride (CCl_4_). There were a total of 24 male Long Evans rats split up into four distinct groups such as control, CCl_4,_ control+cetuximab and CCl_4_+cetuximab. After two weeks of treatment with cetuximab (100 μg/kg), samples of tissue and blood were taken after all the rats had been sacrificed. Plasma samples were examined for the biochemical indicators of inflammation and oxidative stress. Histological staining on liver sections was performed for morphologic pathologies, and related genes expressions analysis were done with RT-PCR in liver tissue. The findings showed that cetuximab could raise the levels of glutathione (GSH), superoxide dismutase (SOD), and catalase (CAT) and considerably lower the levels of aspartate aminotransferase (AST), alanine aminotransferase (ALT), malondialdehyde (MDA), and nitric oxide (NO). Sirius red staining and hematoxylin-eosin (H&E) displayed that cetuximab therapy reduced the inflammatory cells infiltration and enhanced fibrotic lesions. In the meantime, cetuximab therapy also dramatically reduces the expression of genes linked to inflammation in the liver tissue, including NF-кB, iNOS, IL-6, TNF-α, and TGF-β. To sum up, the anti-inflammatory, antifibrotic, and antioxidant properties of cetuximab confer curative efficacy against liver fibrosis.

## Highlights

• Cetuximab, an inhibitor of the EGF receptor (EGFR) prevented the liver fibrosis.
• Cetuximab treatment lowered the anti-inflammatory genes in liver of CCl_4_ administered rats.
• Cetuximab, prevented the oxidative stress and improved liver structure and function in CCl_4_ administered rats.

## 1 Introduction

Liver diseases are among the most common causes of morbidity and death among patients hospitalized in Bangladesh [1]. The three main causes of hepatic disorders are non-alcoholic fatty liver diseases (NAFLD), cirrhosis, and viral hepatitis [2]. According to estimates, between 20% and 30% of people in Western nations have NAFLD [3]. Middle East, Japan, and China also showed similar pattern of the NAFLD diseases prevalence rate same as the Western world, 15–30% [4]. Trend of NAFLD progression in Bangladesh is also increasing because of dietary changes and the sedentary character of modern living among urban population [1,5]. In May 2014, the World Health Organization (WHO) released data showing that 2.82% of deaths in Bangladesh are related to liver disorders. Furthermore, it's estimated that 8 million people have chronic liver complications from infections caused by the hepatitis B and hepatitis C viruses, respectively [6,7].

The hall mark signs for the development and progression of hepatic dysfunction due to virus infection and NAFLD are oxidative stress and inflammation [8,9]. Additionally, the majority of chronic liver illnesses result in hepatic fibrosis, which is defined through an overabundance of proteins found in the extracellular matrix (ECM) [10]. Laboratory animals can develop hepatic dysfunction through a variety of methods, including feeding them alcohol, giving them carbon tetrachloride and diethylnitrosamine (DEN), feeding them a food low in choline, and feeding them a meal high in fat [11,12]. Among them, administration of carbon-tetrachloride is a reliable and reproducible method for the development of hepatic dysfunction in rats [13]. Carbon-tetrachloride metabolism in liver exerts trichlomethyl free radicles which could be involved in the onset of lipid peroxidation, oxidative stress by lowering antioxidant enzymes activities and inflammation in liver. Moreover, carbon-tetrachloride administration also induces hepatic fibrosis if the insult continues.

Numerous studies revealed that the epidermal growth factor receptor (EGFR) is often expressed in the progression of hepatic fibrosis [12,14]. A prior study additionally demonstrated a correlation between greater fibrosis and cirrhosis progression and a variation in the human EGF gene that results in higher EGF production [15,16]. According to a previous study, EGFR inhibition offers high-risk cirrhosis patients a promising treatment strategy for reducing fibrogenesis and preventing hepatocellular carcinoma (HCC) [12]. The transmembrane receptor known as the epidermal growth factor receptor (EGFR) is a part of the family of receptor tyrosine kinases. EGFR can be bound by several ligands, such as transforming growth factor α (TGF-α), epidermal growth factor (EGF), and others [17]. CCl_4_ or thio-acetamide induced chronic toxic injury in livers showed increased HB-EGF and TGF-α expression in the mouse liver [18,19].

Effects of the chimeric monoclonal IgG1 antibody cetuximab, which targets EGFR, in HCC is still unknown, however it significantly slows the growth of many malignancies [20]. A prior study indicates that cetuximab was successful in causing apoptosis and suppressing the cell cycle in HCC cell lines [21]. Report also suggests that by inhibiting EGFR phosphorylation, the small-molecule EGFR inhibitor erlotinib demonstrated a decrease in the quantity of activated HSCs [22]. However, the effect of cetuximab in hepatic fibrosis was not explored and explained to date. In light of the aforementioned research, this study was conducted to assess cetuximab's ability to preserve the liver and its anti-fibrotic properties in rats given CCl_4_.

## 2 Materials and methods

### 2.1 Materials and reagents kits

DCI Diagnostics (Budapest, Hungary) provided the kits for alkaline phosphatase (ALP) (Ref. no. 41257), alanine transaminase (ALT) (Ref. no. 30253), and aspartate transaminase (AST) (Ref. no. 30243). The suppliers of standards, additional reagents for the MDA, NO, APOP, and Picrosirius red staining assays, and Merck (Darmstadt, Germany) were sourced (3050 Spruce St. 63103 St. Louis, USA). Thiobarbituric acid came from the Sigma Chemical Company in St. Louis, Missouri, in the United States. Reduced glutathione (GSH) was purchased from J.I. Baker in the United States. Analytical grade reagents and chemicals were utilized in all other instances. SOD standard and extra components for the analysis were obtained from SR Group located in Delhi, India. DCI Diagnostics (Budapest, Hungary) provided creatinine, uric acid, and creatinine assay kits. RevertAid First Strand cDNA Synthesis Kit and GeneJET RNA Purification Kit (Catalog number: K1621)

### 2.2 Animals and grouping

Four groups of rats (10–12 weeks old, Long Evans, Male, 190–230 g weight, n = 6 rats in each group) were used to examine the hepatoprotective effects of cetuximab.

⁃ Group I (control): For two weeks, animals in Group I received intragastrically administered 0.5 mL/kg of saline (0.85%) and 0.5 mL/kg of olive oil twice a week.
⁃ Group II (CCl_4_): For a period of two weeks, animals in Group II received intragastrically administered 0.5 mL/kg of CCl_4_ (1:3 in olive oil) twice a week.
⁃ Group III (control + cetuximab): For two weeks, subcutaneous administration of cetuximab 100 μg/kg (which was dissolved in PBS) administered daily to the animals in this group.
⁃ Group IV (CCl_4_ + Cetuximab): During two weeks, rats in Group IV received a 0.5 mL/kg dosage of CCl_4_ (1:3 in olive oil) twice a week. Additionally, for two weeks, animals in Group IV received subcutaneous daily injections of cetuximab 100 μg/kg (which was dissolved in PBS).

In this study, male rats were used to minimize the anti-inflammatory effect of estrogen as in female rats which may affect the progression of hepatic inflammation. The previously available literature was used to figure out the dosage of cetuximab, which was 100 μg/kg [23]. The protocols of animal handling and sacrifice of animals for this study were reviewed and approved by the Institutional Animal Care and Use Committee of North South University, Bangladesh (IACUC, NSU) (Approval number- 2022/OR-NSU/IACUC/0308). The IACUC, NSU follows the guideline for animal research prescribed by the Council for International Organization of Medical Sciences (CIOMS) and The International Council for Laboratory Animal Science (ICLAS).

All animals will be put to death with ketamine (80 mg/kg) after two weeks, and the internal organ such as liver as well as their blood were taken. The organs were weighted and kept for future research at −20 °C as soon as they are collected. Using a syringe, blood was extracted, and the samples were centrifuged for 15 min at 4 °C at 8000 rpm. After that, plasma samples were placed in microcentrifuge tubes using a micropipette and kept there at −20 °C, until additional analysis. In assessing cetuximab's hepatoprotective effects, various biological assays for liver function, oxidative stress, inflammation, and oxidative stress associated gene expressions will be carried out in addition to histopathological staining.

### 2.3 Biochemical assays

#### 2.3.1 Liver toxicity assessment

Plasma aspartate aminotransferase (AST), alanine aminotransferase (ALT), and alkaline phosphatase (ALP) activities were measured in order to evaluate the liver function. Using the commercial kit in the Clindiag semi-automatic analyzer, every test was conducted in compliance with the guidelines provided by the manufacturer.

#### 2.3.2 Malondialdehyde (MDA) estimation

MDA concentrations in tissues and plasma were measured in order to evaluate lipid peroxidation. Lipid peroxidation was assayed using a previously described test technique using thiobarbituric reactive substances (TBRAS) [24]. For plasma and tissues, MDA was represented as nmol/mL and nmol/g, respectively.

#### 2.3.3 Estimation of nitric oxide (NO)

As previously stated, the Griess-illosvoy reaction-based assay technique was employed to measure the concentration of NO in tissues and plasma [24,25].

#### 2.3.4 Determination of advanced protein oxidation product (APOP)

Measurements were made of the APOP levels in tissues and plasma [24,26]. Plasma and tissue homogenates were prepared in phosphate buffer solution at a 1:5 ratios and in addition to 0.1 mL KI. The reaction was stopped with the addition of acetic acid after 2 min, and absorbance was measured at 340 nm.

#### 2.3.5 Catalase determination

Catalase is an enzyme that detoxifies H_2_O_2_ produced under oxidative stress. The catalase enzyme activity was ascertained in accordance with a previously described procedure [24,27].

#### 2.3.6 Estimation of SOD enzyme activity

Utilizing a previously outlined approach, the antioxidant enzyme SOD activity was assessed [24,28]. Absorbance was measured while taking into account the suppression of adrenaline auto-oxidation. 480 nm. A control reaction was conducted in addition to the other reaction, yet, absent the sample of tissue or plasma.

#### 2.3.7 Myeloperoxidase (MPO) activity estimation

The MPO activity was evaluated using a reaction technique based on di-anisidine and H_2_O_2_ [27,29]. At 460 nm, the absorption was measured. Protein units expressed as U/min/mg were used for MPO units.

### 2.4 RT-PCR for oxidative stress and inflammation regulatory genes expression

Following sacrifice, the liver tissues of representative animals were obtained while preserving an RNAse-free environment. Utilizing the kit from Thermo-Fisher Scientific, the mRNA was extracted and purified (Massachusetts, USA). Following the NanoDrop 2000 RNA concentration measurement, Thermo-Fisher Scientific's cDNA Synthesis Kit (USA) has been utilized for synthesizing cDNA from 1 μg of mRNA from each of the samples. Thermo Scientific, USA's SYBRTM Green PCR Master Mix and premade primers from the Primer3 website were the tools used to assess the relative expression levels of the mRNA of proteins linked to oxidative stress and inflammation by qRT-PCR in the ΔΔCT method (Table 1). An earlier program was created and authorized by Khan et al. (2016) was used to conduct the quantitative PCR in a CFX96C1000 Touch Real-Time PCR Detection System (Bio-Rad, USA) [30]. The protocol specifies that denaturation at 95 °C, selective annealing at 60 °C, and synthesis at 72 °C be repeated 40 times. CFX ManagerTM Software examined the data in compliance with the recommendations provided by the manufacturer. The control for normalizing the target protein gene expression was thought to be the β-actin transcript level. The primers of genes are given in the Table 1.

**Table-1:** The forward and reverse sequence of the primer which will be applied in this experiment.

Name of gene	Type	Sequence
Nrf-2	Forward	5′-CCC AGCACA TCC AGACAGAC-3′
	Reverse	5′-TATCCAGGGCAAGCGACT C-3′
Heme oxygenase-1 (HO-1)	Forward	5′-TGCTCGCATGAACACTCTG-3′
	Reverse	5′-TCCTCTGTCAGCAGTGCCT-3′
Heme oxygenase-2 (HO-2)	Forward	5′-CACCACTGCACTTTACTTCA-3′
	Reverse	5′-AGTGCTGGGGAGTTTTAGTG-3′
and	Forward	5′-GCTCTAATCACGACCCACT-3′
	Reverse	5′-CATTCTCCCAGTTGATTACATTC-3
Catalase	Forward	5′-ATTGCCGTCCGATTCTCC-3′
	Reverse	5′-CCAGTTACCATCTTCAGTGTAG-3′
Glutathione peroxidase (GPx)	Forward	5′-GGGCAAAGAAGATTCCAGGTT-3′
	Reverse	5′-GGACGGCTTCATCTTCAGTGA-3′
IL-1	Forward	5′-ATGCCTCGTGCTGTCTGACC-3′
	Reverse	5′-CCATCTTTAGGAAGACACGGGTT-3′
IL-6	Forward	5′-AGCGATGATGCACTGTCAGA-3′
	Reverse	5′-GGTTTGCCGAGTAGACCTCA-3′
TNF-α	Forward	5′-ATGTGGAACTGGCAGAGGAG-3′
	Reverse	5′-CCACGAGCAGGAATGAGAAGAG-3′
TGF-β	Forward	5′-AAGAAGTCACCCGCGTGCTA-3′
	Reverse	5′-TGTGTGATGTCTTTGGTTTTGTC-3′
iNOS	Forward	5′-TGGTCCAACCTGCAGGTCTTC-3′
	Reverse	5′-CAGTAATGGCCGACCTGATGTTG-3′
NF-кB	Forward	5′-TGTGAAGAAGCGAGACCTGGAG-3′
	Reverse	5′-GGCACGGTTATCAAAAATCGGATG-3′
β-Actin	Forward	5′-GCGAGAAGATGACCCAGATC-3′
	Reverse	5′-GGATAGCACAGCCTGGATAG-3′

### 2.5 Procedure of histopathology

For several days, liver tissues had been preserved in 10% neutral buffered formalin (NBF). These preserved tissues underwent a graduated xylene treatment before being encased in paraffin wax. After being cut into 5 micron-thick pieces, the paraffin block tissues were put on glass slides. Graded alcohol was used to progressively rehydrate and dehydrate each piece after they had been de-paraffinized with xylene. Hematoxylin and eosin was applied as a final stain to determine the infiltration of inflammatory cells and fundamental tissue architecture. Prussian blue staining shows the presence of free iron, while Sirius red staining indicated the presence of collagen. All photos were taken with a light microscope at 40× magnification [27,31].

### 2.6 Statistical analysis

Mean ± Standard deviation was utilized for each test parameter in the data computation. In this investigation, Graph Pad Prism 9 was used to examine all of the data. A One Way ANOVA and a Tukey test were conducted. All differences were taken into account at *p* ≤ 0.05 for statistical significance.

## 3 Results

### 3.1 Effect of cetuximab on rats treated with CCl_4_ in terms of body weight and liver wet weight

**Fig. 1:**
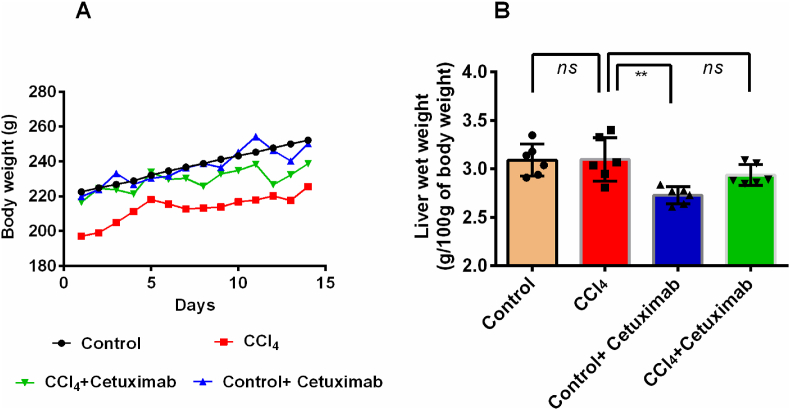
Effect of cetuximab on (A) body weight and (B) liver wet weight in CCl_4_ administered rats. N = 6 and each value was shown as mean ± standard deviation (SD). In the course of the statistical analysis, a Tukey test and one-way ANOVA were conducted. In terms of statistical significance, a value of *p* ≤ 0.05 is deemed significant in every instance. ns in this case denotes p > 0.05.

The results of the investigation showed that in comparison to the control group, rats treated with CCl_4_ exhibited reduced body weights (Fig. 1A). In comparison to the control and CCl_4_ treated groups, respectively, rats in the control + cetuximab and CCl_4_ + cetuximab groups showed an increase in body weight (Fig. 1A). When cetuximab was used to treat CCl_4_-intoxicated rats, the liver wet weight of the treated group did not substantially (*p* ≤ 0.01) decrease from that of the control group, as shown in Fig. 1B. Interestingly, the wet weight of rat liver did not rise noticeably (*p* ≤ 0.01) in pretreated rats receiving cetuximab and CCl_4_ in comparison to rats receiving CCl_4_ treatment (Fig. 1B).

### 3.2 Impact of cetuximab on plasma levels of ALT, AST, and ALP in rats treated with CCl_4_

**Fig. 2:**
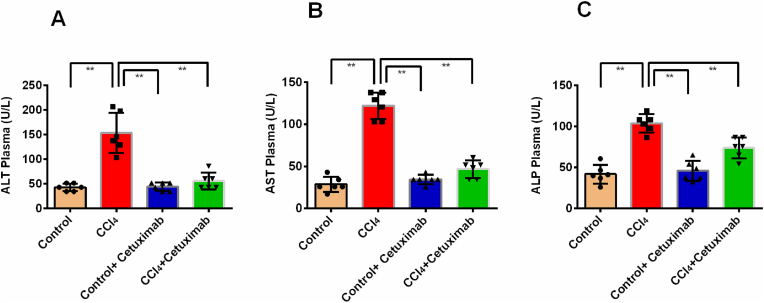
Impact of cetuximab on the (A) ALT, (B) AST, and (C) ALP plasma levels in rats given CCl_4_. The mean ± standard deviation (SD) was used to represent each value. N is equal to 6. A one-way ANOVA and a Tukey test were run as part of the statistical analysis process. In terms of statistical significance, a value of *p* ≤ 0.05 is deemed significant in every instance. Here, *p* > 0.05 is denoted by ns, *p* ≤ 0.05 by *, and *p* ≤ 0.001 by **.

The severity of liver impairment generated by CCl_4_ was increased significantly *(p* ≤ 0.01), when compared to control rats. Rats given CCl_4_ had higher plasma levels of ALP, AST, and ALT (Fig. 2A, B, and 2C). The study examined the impact of cetuximab on various biochemical markers and found that the CCl_4_+ cetuximab group had significantly lower plasma levels of ALT, AST, and ALP than the CCl_4_ intoxicated group (*p* ≤ 0.01) (Fig. 2A, B, and 2C). Furthermore, the plasma ALT, AST, and ALP levels in the control+ cetuximab group considerably normal in comparison to the control rats (Fig. 2A, B, and 2C).

### 3.3 Cetuximab's effect on oxidative stress in rats given CCl_4_

**Fig. 3:**
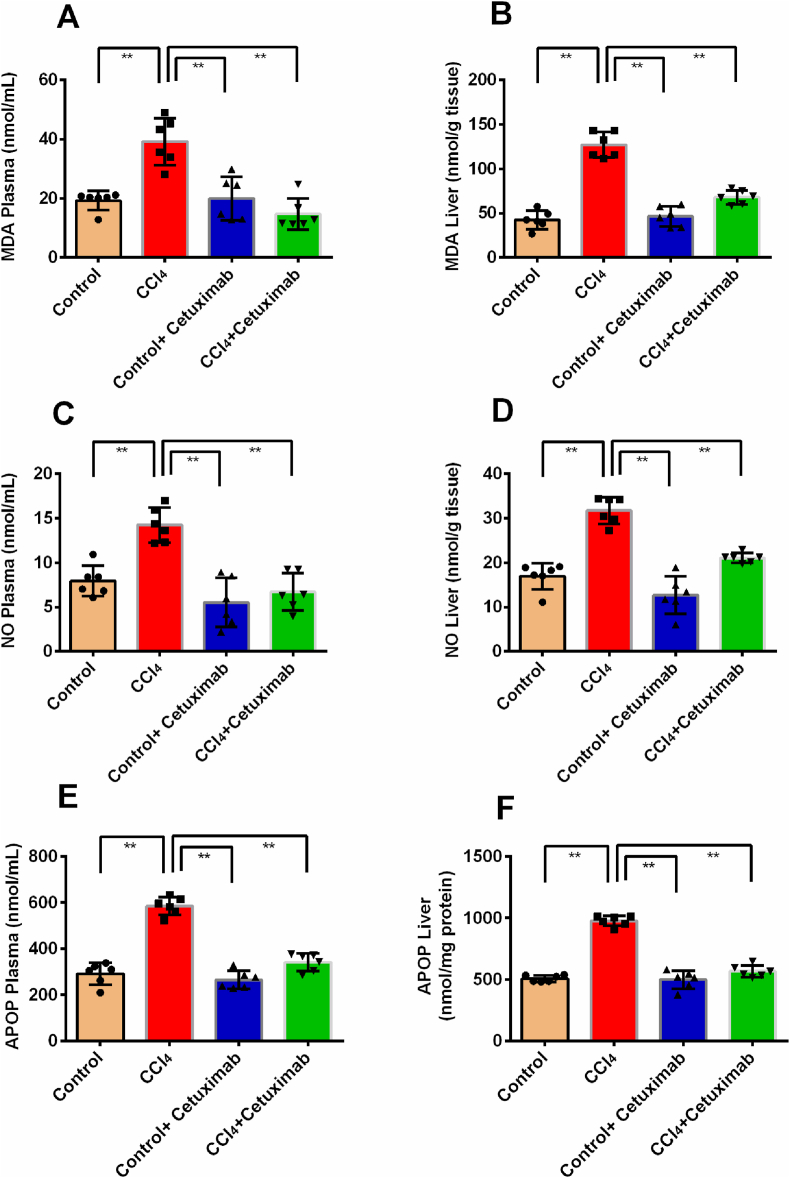
Impact of cetuximab on markers of oxidative stress in rats given CCl_4_. (A) MDA plasma, (B) MDA liver, (C) NO plasma, (D) NO liver, (E) APOP plasma, and (F) APOP liver are shown in this figure. Each value was displayed as mean ± standard deviation (SD), with N = 6. Statistical analysis included running a Tukey test and a one-way ANOVA. In terms of statistical significance, a value of *p* ≤ 0.05 is deemed significant in every instance. Here, *p* ≤ 0.05 by *, and *p* ≤ 0.001 by **.

As seen in Fig. 3A and B, the levels of MDA, a marker of oxidative stress, in the liver and plasma were found to be significantly greater (*p* ≤ 0.01) in the CCl_4_ group compared to the control group. It is important to emphasize that rats receiving cetuximab treatment had significantly lower MDA levels (Fig. 3A and 3B). In comparison to the CCl_4_ group, MDA levels were lower in the control+ cetuximab group (Fig. 3A and B).

In contrast to the control group, the CCl_4_ group's plasma and liver homogenates showed a higher level of the another oxidative stress indicator, NO (*p* ≤ 0.01) (Fig. 3C and D). Rats treated with cetuximab in the CCl_4_+ cetuximab group showed a significant drop in NO levels in the plasma and liver (*p* ≤ 0.01) in comparison to the CCl_4_ group (Fig. 3C and D). When comparing the control+cetuximab group to the CCl_4_ group, there was also a noteworthy reduction in the NO levels in the plasma and liver (Fig. 3C and D).

The CCl_4_ group showed a substantial (*p* ≤ 0.001) increase in the level of APOP in their liver homogenates and plasma when compared with the control group, another crucial oxidative stress marker (Fig. 3E and F). It is important to note that, rats received both CCl_4_ and cetuximab had considerably reduced levels of APOP in plasma and liver homogenates in contrast to the CCl_4_ group (*p* ≤ 0.001) (Fig. 3E and F). Regarding the control+cetuximab group in comparison with the CCl_4_ group, the plasma, liver, and kidney homogenates showed a significant decline in APOP concentration (Fig. 3 E and 3F).

### 3.4 Impact of cetuximab on the activity of antioxidant enzymes in rats given CCl_4_

**Fig. 4:**
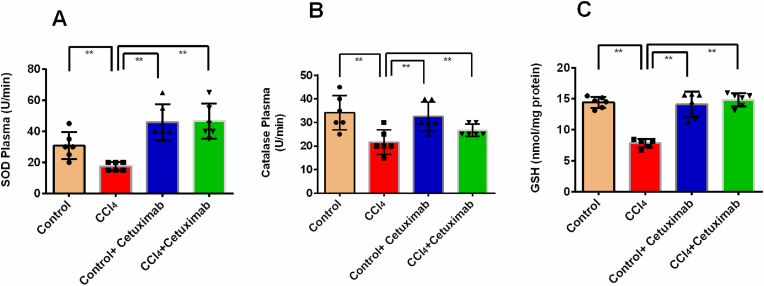
Impact of cetuximab on the activity of antioxidant enzymes in rats given CCl_4_. (A) SOD plasma, (B) Catalase plasma, and (C) GSH plasma are shown in this figure. The mean ± standard deviation (SD) was used to represent each value. N is equal to 6. One-way ANOVA and a Tukey test were run as part of the statistical analysis process. In terms of statistical significance, a value of *p* ≤ 0.05 is deemed significant in every instance. Here, *p* ≤ 0.05 by *, and *p* ≤ 0.001 by **.

Fig. 4A shows the plasma SOD activity of the CCl_4_ group was noticeably reduced (*p* ≤ 0.05) than that of the control rats. After receiving cetuximab treatment, the rats' reduced plasma SOD activity in the CCl_4_-intoxicated group was considerably (*p* ≤ 0.001) recovered (Fig. 4A). Fig. 4A shows that there was a notable increase in SOD activity in the control+cetuximab group (*p* ≤ 0.01) than the CCl_4_ group.

As seen in Fig. 4B, the CCl_4_ group showed significantly (*p* ≤ 0.01) lower plasma catalase activity in a contrast with the control group. The treatment of rats with cetuximab (CCl_4_+ cetuximab) was observed to significantly (*p* ≤ 0.001) raise the catalase activity in plasma (Fig. 4B). In contrast with the CCl_4_ group, the control+cetuximab group exhibited a significantly higher level of catalase activity (Fig. 4B).

GSH is another tissue antioxidant which was restored in the plasma and tissues due to cetuximab treatment. In contrast with rats in the control group, the CCl_4_ group's plasma GSH level was shown to be considerably lower (*p* ≤ 0.05) (Fig. 4C). When rats treated with cetuximab, the decreased plasma GSH level in the CCl_4_-intoxicated group was considerably (*p* ≤ 0.01) recovered (Fig. 4C). By contrast with the CCl_4_ group, the control+cetuximab group similarly showed a substantial increase in GSH levels (*p* ≤ 0.01) (Fig. 4C).

### 3.5 Impact of cetuximab on the expression of antioxidant genes in the liver of rats given CCl_4_

**Fig. 5:**
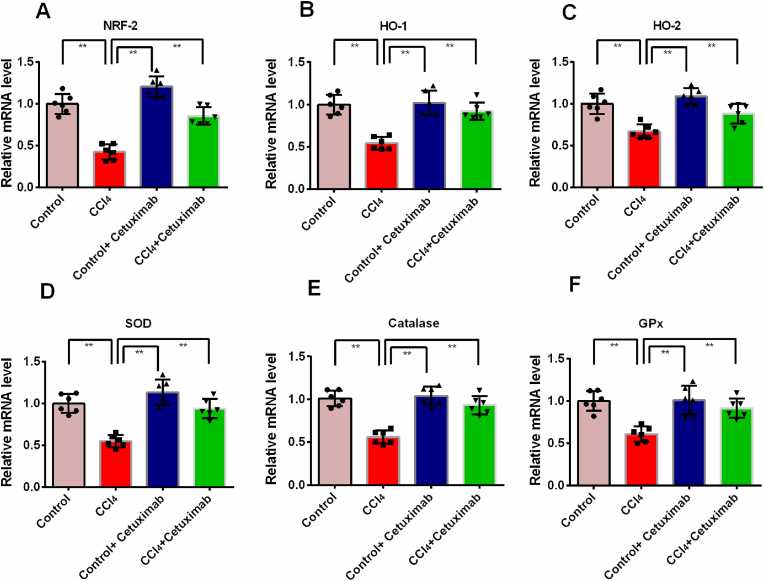
Impact of cetuximab on the expression of genes related to antioxidants in the liver of rats administered CCl_4_. The mean ± standard deviation (SD) was used to represent each value. The mean ± SEM was used to represent each value. N is equal to 6. A one-way ANOVA and a Tukey test were run as part of the statistical analysis process. In terms of statistical significance, a value of p ≤ 0.05 is deemed significant in every instance. Here, *p* ≤ 0.05 by *, and *p* ≤ 0.001 by **.

The expression of genes connected to tissue antioxidants is shown in Fig. 5. The investigation's findings demonstrated that, in comparison to control rats, CCl_4_ treatment dramatically (p ≤ 0.01) reduced Nrf-2 expression in the liver of the rats (Fig. 5A). Rats given CCl_4_ showed increased Nrf-2 expression in their livers after receiving cetuximab treatment (Fig. 5A). According to Fig. 5 B and C, rats treated with cetuximab were able to recover the expression of the HO-1 and HO-2 genes, which had likewise decreased (*p* ≤ 0.01) after receiving CCl_4_ administration. In the liver of rats given CCl_4_, cetuximab therapy keeps the antioxidant enzymes, such as catalase, SOD, and GPx genes, expressed normally (Fig. 5D, E, and F).

### 3.6 Cetuximab's impact on MPO activities in liver of rats given CCl_4_

**Fig. 6:**
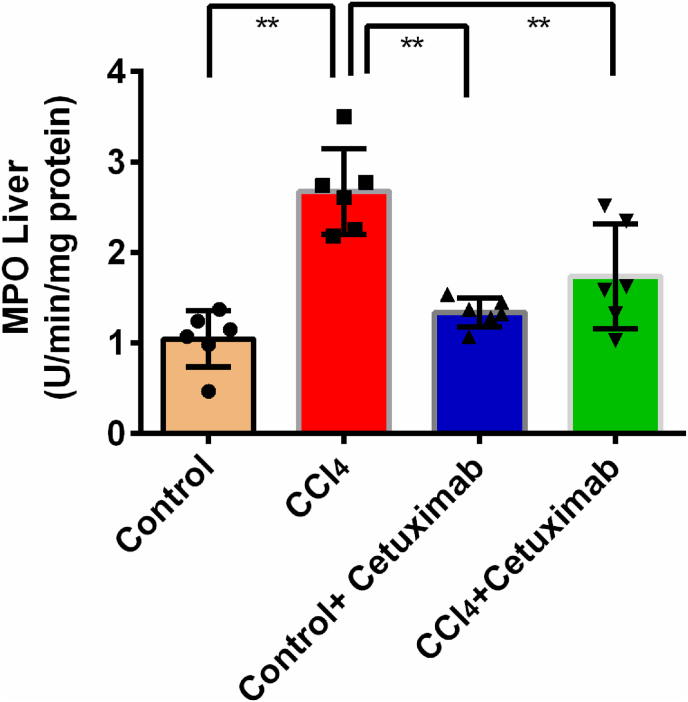
Impact of cetuximab on MPO activity in rats given CCl_4_. For each value, the mean ± standard deviation (SD) was utilized. N is equal to 6. The statistical analysis was carried out employing a Tukey test and one-way ANOVA. Any scenario where *p* ≤ 0.05 is taken into account for statistical significance is considered significant. Here, *p* ≤ 0.05 by *, and *p* ≤ 0.001 by **.

The CCl_4_-intoxicated group's liver MPO activity were found to be markedly (p ≤ 0.01) higher in comparison with the control group (Fig. 6). The administration of cetuximab to the CCl_4_ group (CCl_4_+ cetuximab group) caused the liver's MPO activity to significantly (p ≤ 0.01) decline (Fig. 6). Beyond that, the liver MPO of the control+cetuximab group was higher in comparison to that of the CCl_4_ group activity decreased significantly (*p* ≤ 0.01) (Fig. 6).

### 3.7 Impact of cetuximab on the expression of genes that are associated with inflammation in the liver of rats given CCl_4_

**Fig. 7:**
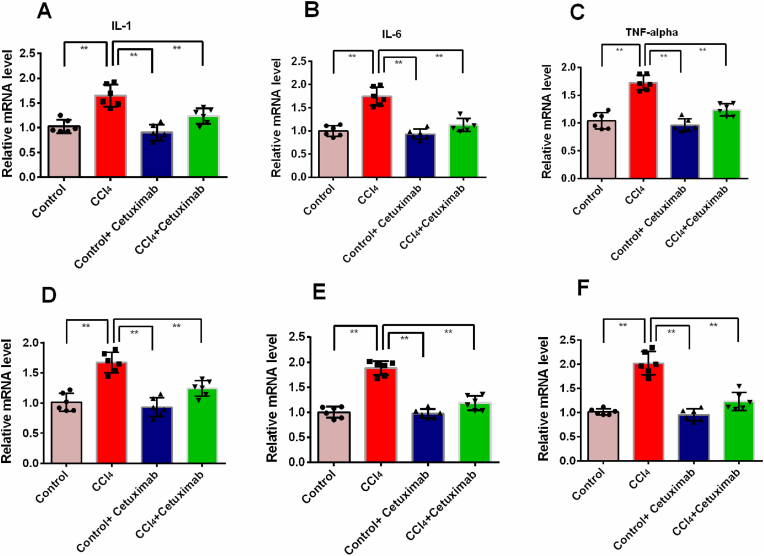
Effectiveness of cetuximab on inflammation-related gene expression in the liver of rats treated with CCl_4_. N = 6, with each value presented as mean ± standard deviation (SD). One-way ANOVA and a Tukey test were run as part of the statistical analysis process. In terms of statistical significance, a value of *p* ≤ 0.05 is deemed significant in every instance. Here, *p* ≤ 0.05 by *, and *p* ≤ 0.001 by **.

The expression of inflammatory cytokines in the liver is depicted in Fig. 7. The related mRNA expression of TNF-α, IL-1, and IL-6 considerably (*p* < 0.01) raised in the liver of rats treated with CCl_4_ (Fig. 7A, B, and C). When cetuximab was given to the CCl_4_ group (cetuximab + CCl_4_ group), TNF-α, IL-6, and IL-1 expression were all considerably (*p* ≤ 0.01) decreased (Fig. 7 A, B, and C).

Rats administered with CCl4 exhibited elevated TGF-β expression in their livers in contrast to the control group (Fig. 7D). Rats given CCl_4_ showed normalized TGF-β expression in their livers after receiving cetuximab treatment (Fig. 7D). Rats administered with CCl_4_ also exhibited greater hepatic expression of iNOS and NF-кB in contrast with the control group (Fig. 7E and F). Rats given CCl_4_ had normalized NF-кB and iNOS expression in their livers following cetuximab treatment (Fig. 7E and F).

### 3.8 Impact of cetuximab on the histopathology of the liver in rats given CCl_4_

**Fig. 8:**
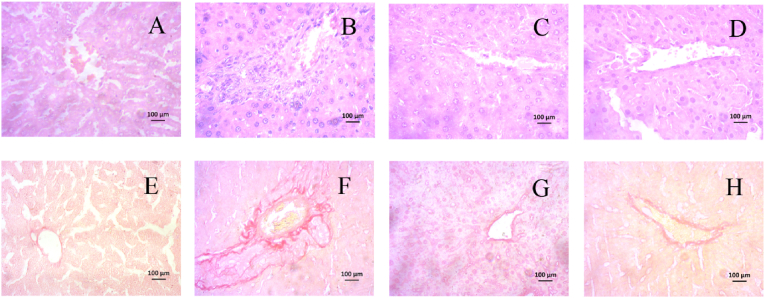
Effectiveness of cetuximab on the liver of rats' histology after CCl_4_ administration. Hematoxylin and eosin staining is shown in this picture from A to D, while Sirius red staining is shown from E to H. Here, A, E-Control; B, F- CCl_4_; C, G- Control+cetuximab; and D, H- CCl_4_+cetuxibmab. Each picture was captured with a 40× magnification.

Fig. 8 present the results of liver histology in rats treated with cetuximab. The findings showed that there was no hepatic inflammation or collagen deposition in the liver of the control group of rats (Fig. 8A–and E). The CCl_4_-treated group, on the other hand, showed significant collagen deposition and hepatic inflammation (Fig. 8C and F). The lobule and hepatocyte morphology and orientation were normal in the control+cetuximab group (Fig. 8B). It was discovered that in rats, pretreatment with cetuximab reduced inflammation, decreased collagen deposition, and repaired hepatic necrosis. (Fig. 8D and H).

## 4 Discussion

One significant regulator of the onset of fibrosis in several organs, such as the liver, is the epidermal growth factor (EGF). This study showed that in rats given CCl_4_, the EGF-receptor inhibitor cetuximab abled to stop liver damage and fibrosis. Cetuximab showed protective effects because it may stop the expression of genes linked to inflammation in the liver and activate antioxidant enzymes to scavenge the production of free radicals caused by CCl_4_. CCl_4_ is a noxious chemical agent mostly used now a day for the emergence of experimental fibrosis and hepatic damage in rodents [32]. The hallmark of CCl_4_ administered liver damage is the increment of transaminases activities in plasma. The damaged hepatocytes release the ALT, AST, and ALP enzymes in the extracellular fluids and showed their increased activities. Any protection of the damaged liver starts lowering these enzymes activities. The findings of this research showed that rats treated CCl_4_ had higher AST, ALT, and ALP enzymes activity while their levels were decreased by the EGFR inhibitor cetuximab treatment at a dose of 100 μg/kg. Prior reports that provided evidence for this conclusion revealed that EGFR inhibition may protect the liver injury in various experimental animal models [33,34].

CCl_4_ is mainly metabolized in liver microsomal enzymes cytochrome-P450 and may convert into tri-chloromethyle free radicles [32]. Free radicle production may disrupt the cellular organelle and rupture the cell membrane by oxidation. Hepatic cell damage due to free radicles may increase the creation of the end result of lipid peroxidation, mostly malondialdehyde (MDA) in liver and initiate a condition called oxidative stress [35]. Rats given CCl_4_ treatment had higher MDA levels in their liver and plasma, along with higher levels of nitric oxide (NO) and advanced protein oxidation product (APOP), two other indicators of oxidative stress [35]. Rats given CCl_4_ showed an increment in those oxidative stress-related indicators in their liver could be a direct result of declined antioxidant enzymes activities (catalase and SOD) both in plasma and liver [35]. Epidermal growth factor receptor (EGFR) is tyrosine kinases family proteins which are responsible for the growth, proliferation, and differentiation of various cells including liver cells [36]. Previous report suggests that EGFR expression stimulates the production of hydrogen peroxide through NADPH-oxidase system and participates in the development of oxidative stress [37]. CCl_4_ administration showed increased EGFR expression in liver of rats which aggravates the liver injury [38]. Treatment with the EGFR inhibitor cetuximab in rats given CCl_4_ demonstrated lower levels of MDA in the liver and plasma, in conjunction with lower levels of NO and APOP, which are found in this study.

The tissue antioxidants are regulated by the genetic expression of Nrf-2-HO-1 mediated pathway [39]. In order to treat liver diseases like alcoholic liver disease (ALD), non-alcoholic steatohepatitis (NASH), viral hepatitis, non-alcoholic fatty liver disease (NAFLD), hepatocellular carcinoma (HCC), hepatic ischemia-reperfusion injury (IRI), and, acute liver failure, it is currently believed that the Nrf-2 mediated pathway is a key therapeutic strategy [39,40]. High level of Nrf-2 expression has been observed in liver and kidney tissues as these are the most metabolically active tissues [40]. Free radicals and oxidative stress may trigger Nrf-2-mediated signaling, which controls genes expression of enzymes that protect against oxidative damage like SOD, catalase, GPX, and others. This signaling is followed by the activation of HO-1 [41]. Previous report suggests that the declined tissues antioxidant defense may be restored in CCl_4_ administered rats partly by activation of Nrf2/HO-1 [42]. The antioxidant enzyme activities in this study, including SOD, catalase, and GSH levels were declined in CCl_4_ administered rats followed by a declined in antioxidant enzymes gene expressions in the liver. EGFR inhibitor cetuximab treatment at (100 μg/kg) also restored the antioxidant enzymes such as catalase, SOD and GSH in liver and plasma of CCl_4_ administered rats. The restoration of antioxidant genes was partly mediated through the Nrf-2 –HO-1 expression in the liver. This outcome is further corroborated by earlier research that demonstrated high fat diet may decline the Nrf-2 –HO-1 expression which was restored by EGFR inhibitor [22].

Administering CCl_4_ to rats also triggers an inflammatory event in their livers. This investigation revealed that rats given CCl_4_ had much higher liver MPO activity, which was subsequently reduced by cetuximab treatment. MPO is associated with immune cells [43]. Previous report suggests that MPO is expressed highly in CCl_4_ administered rats's liver [43]. It is evident from this study that mononuclear inflammatory cells are infiltrated in CCl_4_ administered rats's liver. In rats given CCl_4_, cetuximab therapy also abolished the infiltration of inflammatory cells and necrosis. Furthermore, the genes involved in inflammation, including iNOS, TNF-alpha, IL-1, and IL-6 are significantly lowered by cetuximab treatment in CCl_4_ administered rats. This discovery aligns with earlier research that shown EGFR inhibitor may decline the inflammatory cytokines expression *in vitro* [44].

Oxidative stress and inflammation may influence the development of fibrosis in the necrotized region of liver due to CCl_4_ administration. This study demonstrated that exposure to CCl_4_ caused liver fibrosis in rats. Previous report also showed the EGFR expression is involved in the emergence of CCl_4_-administered liver fibrosis occurs most likely through activating macrophages and hepatic stellate cells [45]. Moreover, oxidative stress and inflammatory mediators could trigger the hepatic stellate cells (HSCs) to secrete collagen, the primary extracellular matrix protein [46]. This fibrogeneic response is further stimulated by the TGF-beta mediated signaling which stimulates the fibroblast cells to stimulate collagen deposition [47,48]. When rats were given CCl_4_, their livers expressed more inflammatory genes and TGF-beta gene expression together [35,49]. EGFR inhibitor has been demonstrated EGFR phosphorylation-mediated HSC inactivation and prevented fibrosis in rats [12]. Furthermore, EGFR inhibition (Small-molecule inhibitors: AG1478 and compound 451) may have also stopped the fibrosis that occurred in the livers of male C57/BL6 mice on a high-fat diet [22]. In this study, EGFR inhibitor cetuximab treatment in CCl_4_ given rats decreased the TGF-beta gene expression and signaling, which in turn, prevented the development of fibrosis and collagen buildup in the liver of rats.

In conclusion, this research work provides evidences that cetuximab treatment may inhibit the inflammation mediated fibrosis in CCl_4_ administered rats. Both the restoration of antioxidant enzymes and the halting of inflammatory gene expression in the hepatocytes may be responsible for the protective effect. Further, investigation is warranted to know the high dose effect of cetuximab in liver as well as on the effect of human hepatic disorder.

## Funding

The investigators acknowledge the generous funding from the Ministry of Science and Technology, Bangladesh under Special Allocation Grant to Dr. Nusrat Subhan., with all logistical backing and lab facilities provided by the North South University Department of Pharmaceutical Sciences.

## Ethical statement

The protocols of animal handling and sacrifice of animals for this study were reviewed and approved by the Institutional Animal Care and Use Committee of North South University, Bangladesh (IACUC, NSU) (Approval number- 2022/OR-NSU/IACUC/0308).

## CRediT authorship contribution statement

**Mirza Alimullah:** Writing – original draft, Methodology, Investigation, Formal analysis, Data curation. **Asif Ul Haque Shuvo:** Writing – original draft, Methodology, Investigation, Formal analysis, Data curation. **Ishrat Jahan:** Writing – original draft, Methodology, Investigation, Formal analysis. **Iffat Nowshin Ismail:** Software, Methodology, Investigation, Formal analysis, Data curation. **S.M. Mufidul Islam:** Software, Methodology, Investigation, Formal analysis. **Mahnaj Sultana:** Methodology, Investigation, Formal analysis. **Mahmudur Rahman Saad:** Project administration, Investigation, Formal analysis, Data curation. **Sabbir Raihan:** Writing – original draft, Visualization, Investigation, Formal analysis. **Ferdous Khan:** Writing – review & editing, Writing – original draft, Supervision, Methodology, Investigation, Formal analysis, Conceptualization. **Md. Ashraful Alam:** Writing – review & editing, Writing – original draft, Supervision, Project administration, Data curation, Conceptualization. **Nusrat Subhan:** Writing – review & editing, Writing – original draft, Supervision, Project administration, Investigation, Funding acquisition, Data curation, Conceptualization.

## Declaration of competing interest

The authors declare that they have no known competing financial interests or personal relationships that could have appeared to influence the work reported in this paper.

## Data Availability

Data will be made available on request.
